# Early outcomes following Duramesh™ implantation for abdominal wall closure: the Croydon experience

**DOI:** 10.3389/fsurg.2026.1918733

**Published:** 2026-09-03

**Authors:** Lawrence Nip, Sarah Zhao, Sajid Malik, Reif Hwang, Jessica Quimpo, Eslam Ghazy, Matt Dunstan, Rhys Thomas, Samuel G. Parker

**Affiliations:** 1 The Abdominal Wall Unit, Croydon University Hospital, London, United Kingdom; 2 Division of Medicine, Centre for Medical Imaging, the Rayne Institute, University College London, London, United Kingdom

**Keywords:** Duramesh, incisional hernia, mesh suture, umbilical hernia repair, ventral hernia

## Abstract

**Introduction:**

Mesh suture, or Duramesh™, is gaining traction for abdominal wall closure and ventral hernia repair. However, published data are limited to US case series and institutional registries. Use in UK and European cohorts remains undocumented. This is the first UK series describing indications and outcomes following Duramesh implantation.

**Methods:**

This is a retrospective case series from a medium-sized tertiary abdominal wall unit in the UK. Institutional implant logs of consecutive patients from 1st Sep 2023 to 8th June 2025 were cross-examined to identify relevant patients. Chart review was performed and the indications and outcomes analysed using descriptive statistics. Subgroups were compared with chi-squared and Mann–Whitney *U* tests.

**Results:**

In total, 73 patients were included in this series, with a median follow-up of 227 days. Indications for Duramesh implantation included ventral hernia repair (*n* = 63; 86%) and abdominal wall closure (*n* = 10; 14%). Regarding ventral hernias, the most common location was M3 (*n* = 47; 76%) and median defect size was 2 cm. Planar mesh was used concurrently in 22% (*n* = 16) of cases. The 90-day surgical site occurrence rate was 22%. There were no hernia occurrences or recurrences. Hernias repaired using Duramesh as the sole implant were smaller (*p* = 0.000001) than those repaired using both Duramesh and planar mesh.

**Conclusion:**

There is ongoing debate about whether to repair simple and small midline ventral hernias with planar mesh or suture. Our short-term outcomes suggest Duramesh is safe in this cohort and can be technically convenient in certain situations.

## Introduction

Over the past several decades, substantial research has focused on techniques for abdominal wall closure and ventral hernia repair. Despite this, incisional hernia remains a significant and largely unsolved clinical problem. Primary closure of laparotomy using sutures alone is associated with hernia rates of approximately 9%–20% at 1 year (1, 2), with the rate of recurrence increasing after each subsequent repair (3). The use of mesh has been widely adopted to mitigate recurrence (4). However, there are still scenarios where surgeons remain hesitant to implant mesh such as gross contamination in the presence of a complex hernia, prophylactic mesh implantation following primary laparotomy and small ventral hernias with defects <1–2 cm. For this manuscript, we focus predominantly on the latter. In such cases, suture repair is sometimes preferred where the perceived risk or time required for mesh placement outweighs the benefit of reducing recurrence (5).

**Figure 1:**
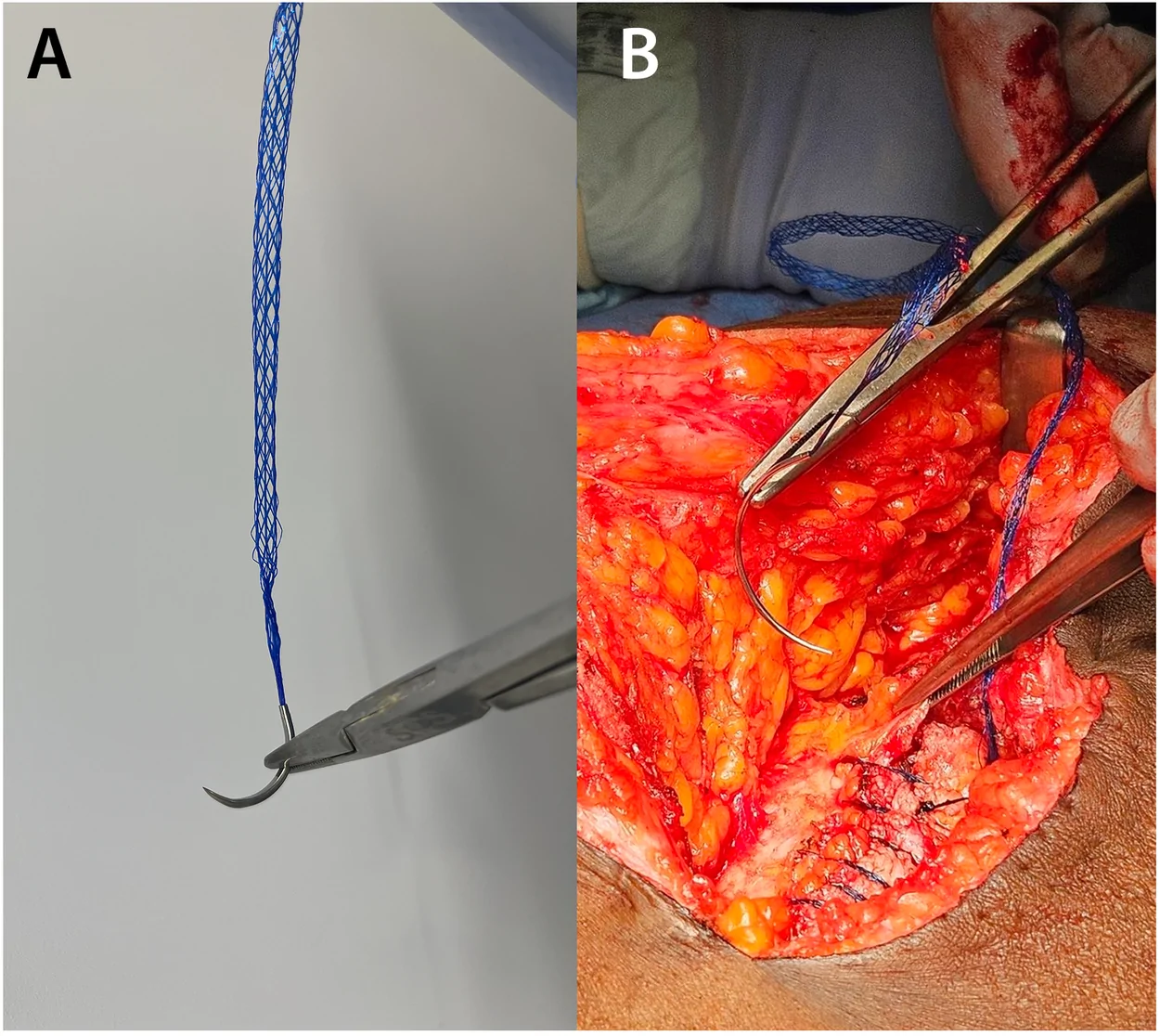
**(A)** Laboratory photograph showing Duramesh MSP500 outside of its packaging. **(B)** Clinical photograph showing Duramesh MSP301 being used for abdominal wall closure.

Duramesh™ represents a novel alternative that seeks to bridge the gap between traditional suture and planar mesh (Figures 1A,B). Composed of synthetic polypropylene filaments woven into a porous, hollow cylinder attached to a curved needle, it is handled and secured like a conventional suture. The design allows it to pass through tissue relatively smoothly, but when tension is applied during knotting, it collapses into a ribbon of mesh around the incision (6). There is growing evidence that early wound failure and partial separation of the fascia lead to incisional hernia formation (7, 8). Duramesh aims to solve this by reducing the risk of “cheesewiring,” or “pull-through”, a phenomenon in which sutures cut through tissue under excessive tension. Additionally, its macroporous architecture permits tissue ingrowth between filaments, analogous to traditional mesh, as supported by histological analyses (9).

Duramesh has been in clinical use in the UK and Europe since receiving CE marking in May 2021. Our previous systematic review indicates that Duramesh appears to be safe and associated with low recurrence rates (10). However, the available literature is largely confined to retrospective series from North American cohorts (11). Consequently, there is a need for further evaluation in broader international populations. Whilst it is licensed for use in abdominal wall closure and ventral hernia repair, these indications remain generic and the optimal clinical applications have yet to be clearly defined. The present study aims to evaluate early outcomes following Duramesh implantation and to clarify the clinical scenarios for which our institution has seen the greatest benefit. Based on our literature search (10), this is the first published UK Duramesh case series.

## Methods

### Study design

This is a single-centre retrospective case series conducted at a medium-sized tertiary abdominal wall reconstruction (AWR) referral centre serving a population of approximately 400,000. The service is co-led by the senior authors who bear the greatest AWR workload, with additional input from 10 consultant surgeons who perform hernia procedures and utilise Duramesh on a case-by-case basis.

Ethical approval was not required under the UK Health Research Authority (HRA) framework, which exempts data collected during routine clinical care for service evaluation. Given emerging evidence that Duramesh may be associated with low rates of recurrence, surgical site occurrence (SSO) and technical ease of implantation (12), it has been used at surgeon discretion since September 2023. All consultant surgeons are regular attendees of the local multidisciplinary team (MDT) meeting where mesh reinforcement options including Duramesh are specifically addressed. Hence, the project was registered locally as a service evaluation and conducted in line with institutional clinical governance processes.

### Patients

Institutional implant logs from both elective and emergency general surgery theatres were retrospectively reviewed to identify eligible patients. All consecutive patients aged 18 years and older who underwent Duramesh implantation for abdominal wall closure or ventral hernia repair between 1st September 2023 and 8th June 2025 were included. We excluded cases where Duramesh was used outside the ventral abdomen. In the elective setting, patients were specifically consented beforehand for implantation of synthetic mesh material. In the emergency setting, all patients were consented for hernia repair with an option to proceed to mesh implantation as required. Owing to the retrospective design, patient selection for Duramesh was at the discretion of the operating consultant guided by MDT outcome or internal departmental review.

### Technique

All surgeons at the local institution were aware of the following guidance based on the manufacturer's instructions. Bites of Duramesh should be taken 8 mm from the fascial edge and spaced at 8 mm intervals. Implantation method can be continuous or interrupted based on surgeon preference. Positioning and tension should be checked with every bite to avoid tissue strangulation. Upon completion of closure, a standard reef knot with four to six throws is secured. The knot may be buried at surgeon discretion in patients with low body mass index or in situations where there is concern about mesh suture prominence through the skin. This can be performed by cutting one of the tails and taking a further bite into the subcutaneous fat to invert the knot. If not buried, both tails are cut to approximately 3 mm in length.

### Outcomes

The primary outcome was the incidence of surgical site occurrence (SSO) within 90 days, initially defined according to the Centers for Disease Control and Prevention criteria and subsequently modified by Majumder et al. (13). Secondary outcomes included surgical site infection (SSI), hernia occurrence or recurrence at the operative site, medical complications (not wound related), 90-day readmission, 90-day reoperation, 90-day mortality, and length of hospital stay. Hernia occurrence or recurrence was defined as a new symptomatic hernia determined by clinician assessment during outpatient follow-up and/or radiological evaluation, when available. Duration of follow-up was measured from the operative date to the most recent outpatient encounter. All patients had at least a telephone consultation and if asymptomatic, were deemed not to have hernia occurrence/recurrence.

### Data collection

Data were extracted from the Trust electronic clinical documentation system (PowerCharts, CernerWorks, Oracle Health, Missouri, United States). Records reviewed included clinical notes, operative notes, anaesthetic records, imaging reports, outpatient documentation, and clinical correspondence. Retrospective data were entered into Microsoft Excel (Microsoft Corporation, Washington, USA) and stored on a secure Trust network server. Variables collected included baseline patient demographics, operative characteristics, and postoperative outcomes as defined above. Where applicable, hernias are characterised according to the European Hernia Society (EHS) classification which is defined by midline (M) and lateral (L) zones and craniocaudal distances from the umbilicus (1–5) (14). In addition, hernias are further defined as primary, incisional or recurrent incisional.

### Statistical analysis

Statistical analysis was performed using Microsoft Excel (Microsoft Corporation, Washington, USA). Descriptive statistics were used to summarise patient characteristics, operative details, and outcomes. Dichotomous variables are presented as numbers and percentages, while continuous variables are reported as medians with inter-quartile ranges (IQR). Subgroup analysis was performed for patients with ventral hernias, split into those which were repaired with Duramesh as the sole implant or used concurrently with planar mesh. Patient demographics, hernia characteristics and outcomes were compared between subgroups. All data were assumed to follow non-parametric distributions. Hence, the chi-squared and Mann Whitney statistics were utilised for dichotomous and continuous variables respectively. A *p*-value of <0.05 was considered statistically significant.

## Results

### Entire cohort

In total, 75 patients were implanted with Duramesh during the study period, of which 2 inguinal hernia repairs were excluded. Patient demographics and operative characteristics of the remaining 73 cases are shown in Table 1. 100% of patients reached 90-day follow-up, with a median follow-up of 227 days (IQR 253). Duramesh was implanted for 3 surgical indications, namely ventral hernia repair (86%), ostomy site closure (8%) and laparotomy closure (6%). In terms of contamination grade, 73% of operations were clean (CDC grade 1) whilst 27% were in clean-contaminated or contaminated fields (CDC grades 2–4). Four-fifths were elective cases (79%), and one fifth were emergency cases (21%).

**Table 1:** Demographic and operative characteristics of the study population.

	All patients	Subgroup 1: VHR with Duramesh as sole implant	Subgroup 2: VHR with Duramesh and planar mesh	Subgroup 3: Ostomy site and laparotomy closure	*P* Value between subgroups 1 & 2
Value (median with IQR or *n* with %)	*n* = 73	*n* = 47	*n* = 16	*n* = 10	*P*-value
Demographics					
Age (years)	58 (23)	54 (23)	61 (20)	74 (15)	0.49
BMI (kg/m^2^)	30 (11)	30 (9)	35 (7)	27 (4)	0.075
Female sex	32 (44%)	23 (49%)	6 (38%)	3 (30%)	0.43
Active smokers	10 (14%)	8 (17%)	1 (6%)	1 (10%)	0.29
Diabetes	15 (21%)	8 (17%)	5 (31%)	2 (20%)	0.22
COPD	1 (1%)	1 (2%)	0 (0%)	0 (0%)	0.56
Previous abdominal surgery	42 (57%)	21 (45%)	12 (75%)	9 (90%)	0.036*
ASA grade					
ASA 1	8 (11%)	8 (17%)	0 (0%)	0 (0%)	—
ASA 2	35 (48%)	22 (47%)	12 (75%)	1 (10%)	—
ASA 3	27 (37%)	16 (34%)	4 (25%)	7 (70%)	—
ASA 4	3 (4%)	1 (2%)	0 (0%)	2 (20%)	—
Operative characteristics					
Surgical indications					
Ventral hernia	63 (86%)	47 (100%)	16 (100%)	0 (0%)	—
Ostomy site closure	6 (8%)	0 (0%)	0 (0%)	6 (60%)	—
Laparotomy mass closure	4 (6%)	0 (0%)	0 (0%)	4 (40%)	—
Urgency					
Elective	58 (79%)	34 (72%)	15 (94%)	9 (90%)	0.075
Emergency	15 (21%)	13 (28%)	1 (6%)	1 (10%)	0.075
CDC Grade					
1	53 (73%)	39 (83%)	13 (81%)	1 (10%)	—
2	10 (14%)	2 (4%)	0 (0%)	8 (80%)	—
3	8 (11%)	6 (13%)	2 (13%)	0 (0%)	—
4	2 (3%)	0 (0%)	1 (6%)	1 (10%)	—
Duramesh implant					
MSP300	55 (75%)	43 (91%)	6 (38%)	6 (60%)	0.000007*
MSP301	18 (25%)	4 (9%)	10 (62%)	4 (40%)	0.000007*
Ventral hernia characteristics (*n* = 63; *n* = 47; *n* = 16; *n* = 0 respectively)					
Defect width (cm)	2 (3)	1.5 (1.5)	5 (2.5)	—	0.000001*
Hernia location					
Midline	57 (92%)	47 (100%)	10 (63%)	—	0.00001*
M1	1 (2%)	1 (2%)	0 (0%)	—	—
M2	7 (11%)	7 (15%)	0 (0%)	—	—
M3	47 (75%)	38 (81%)	9 (56%)	—	—
M4	1 (2%)	0 (0%)	1 (6%)	—	—
M5	1 (2%)	1 (2%)	0 (0%)	—	—
Lateral	6 (10%)	0 (0%)	6 (38%)	—	0.00001*
L2	3 (5%)	0 (0%)	3 (19%)	—	—
L3	3 (5%)	0 (0%)	3 (19%)	—	—
Hernia type					
Primary hernia	38 (60%)	33 (70%)	5 (31%)	—	—
Incisional	17 (27%)	9 (19%)	8 (50%)	—	—
Recurrent incisional	8 (13%)	5 (11%)	3 (19%)	—	—

VHR, ventral hernia repair; IQR, inter-quartile range.

* *p* < 0.05 and therefore represents a significant difference between subgroups on *X*^2^ or Mann–Whitney analysis.

Two types of Duramesh were used: MSP300-5 (26 mm needle) in 75% and MSP301-5 (48 mm needle) in 25%. The mesh suture component is equivalent to number 1 Prolene and is the same in both products (the main difference is needle size). Median operative time was 68 min (IQR 77).

Outcomes are shown in Table 2. The 90-day SSO rate was 22% (*n* = 16), occurring in 10 patients (14%). The 90-day SSI rate was 10% (n = 7), occurring in 6 patients (8%). There were 3 (4%) superficial infections, 3 (4%) deep wound infections and 1 (1%) organ space infection. Regarding other wound events, there were 3 (4%) haematomas, 1 (1%) seroma, 4 (5%) instances of superficial wound dehiscence and 1 (1%) suture granuloma which later required Duramesh explant at 196 days. There were no incidences of fascial dehiscence or enterocutaneous fistulae. There were no hernia occurrences or recurrences.

**Table 2:** Outcomes following Duramesh implantation.

	All patients	Subgroup 1: VHR with Duramesh as sole implant	Subgroup 2: VHR with Duramesh and planar mesh	Subgroup 3: Ostomy site and laparotomy closure	*P* Value between subgroups 1 & 2
Value (median with IQR or *n* with %)	*n* = 73	*n* = 47	*n* = 16	*n* = 10	*P*-value
General outcomes					
Length of follow up (days)	227 (253)	218 (234)	386 (161)	107 (48)	0.024*
Operative duration (mins)	68 (77)	53 (31)	125 (115)	147 (108)	0.000007*
Length of hospital stay	1 (5)	0 (2)	5 (8)	7 (6)	0.009*
Complications					
Surgical site occurrence (SSO)	16 (22%) in 10 (14%) patients	4 in 3 (6%) patients	7 in 3 (19%) patients	5 in 4 (40%) patients	0.15
Surgical site infection (SSI)	7 (10%) in 6 (8%) patients	1 in 1 (2%) patient	4 in 3 (19%) patients	2 in 2 (20%) patients	0.019*
Superficial	3 (4%)	1 (2%)	0 (0%)	2 (20%)	0.56
Deep	3 (4%)	0 (0%)	3 (19%)	0 (0%)	0.002*
Organ space	1 (1%)	0 (0%)	1 (6%)	0 (0%)	0.08
Surgical site event (SSE)	9 (12%) in 9 (12%) patients	3 in 3 (6%) patients	3 in 3 (19%) patients	3 in 3 (30%) patients	0.15
Haematoma	3 (4%)	1 (2%)	1 (6%)	1 (10%)	0.42
Seroma	1 (1%)	1 (2%)	0 (0%)	0 (0%)	0.56
Wound dehiscence	4 (5%)	0 (0%)	2 (13%)	2 (20%)	0.014*
Fascial dehiscence	0 (0%)	0 (0%)	0 (0%)	0 (0%)	1
Enterocutaneous fistula	0 (0%)	0 (0%)	0 (0%)	0 (0%)	1
Suture granuloma	1 (1%)	1 (2%)	0 (0%)	0 (0%)	0.56
Hernia occurrence or recurrence	0 (0%)	0 (0%)	0 (0%)	0 (0%)	1
Medical complication (non-AWR)	19 (26%) in 15 (21%) patients	4 in 4 (25%) patients	4 in 3 (19%) patients	11 in 8 (80%) patients	0.84
Pneumonia	2 (3%)	1 (2%)	1 (6%)	0 (0%)	0.42
Urinary tract infection	3 (4%)	1 (2%)	0 (0%)	2 (20%)	0.56
Electrolyte derangement	5 (7%)	0 (0%)	0 (0%)	5 (50%)	1
Ileus	3 (4%)	0 (0%)	1 (6%)	2 (20%)	0.084
Respiratory failure	2 (3%)	2 (4%)	0 (0%)	0 (0%)	0.40
Acute kidney injury	1 (1%)	0 (0%)	1 (6%)	0 (0%)	0.40
Anaemia requiring blood transfusion	1 (1%)	0 (0%)	0 (0%)	1 (10%)	1
Arrhythmia	2 (3%)	0 (0%)	1 (6%)	1 (10%)	0.084
Other outcomes					
Mortality (90 day)	1 (1%)	1 (2%)	0 (0%)	0 (0%)	0.56
Readmission (90 day)	2 (3%)	1 (2%)	1 (6%)	0 (0%)	0.42
Reoperation (90 day)	1 (1%)	0 (0%)	1 (6%)	0 (0%)	0.084
Duramesh explantation	1 (1%)	1 (2%)	0 (0%)	0 (0%)	0.56

VHR, ventral hernia repair; IQR, inter-quartile range.

* *p* < 0.05 and therefore represents a significant difference between subgroups on *X*^2^ or Mann–Whitney analysis.

Other medical (non-AWR) complications are also reported in Table 2. There was 1 mortality within 90 days, unrelated to the device but rather as a result of respiratory failure in a comorbid patient who underwent emergency umbilical hernia repair. There were 2 readmissions within 90 days. One patient developed a haematoma measuring 16 cm × 11 cm × 3 cm anterior to the retrorectus mesh following abdominal wall reconstruction, which resolved with conservative management. The other readmission was unrelated to the index operation and was due to a separate presentation of testicular pain (with suspicion of epididymoorchitis) 2 weeks after supraumbilical hernia repair.

### Subgroup analysis

Of the 73 patients, 63 (86%) underwent ventral hernia repair. The most common location using the EHS classification was M3 (75%), followed by M2 (11%). L2 and L3 hernias represented 5% of the cohort each. M1, M4 and M5 hernias represented 2% of the cohort each. With regards to hernia type, it was primary in 60%, incisional in 27% and recurrent incisional in 13%. The median defect size was 2 cm (IQR 3).

Duramesh was used as the sole implant for ventral hernia repair in 75% (*n* = 47) and used concurrently with planar mesh in 25% (*n* = 16). The planar meshes used in descending order of frequency were OviTex core (TelaBio; *n* = 6; 38%), Prolene (Ethicon; *n* = 5; 31%), OviTex 1s (TelaBio; *n* = 3; 19%), Ventralex (Becton Dickinson; *n* = 1; 6%) and Progrip (Covidien; *n* = 1; 6%). Duramesh as a sole implant was used to repair smaller hernias compared to when used concurrently with planar mesh (median defect width 1.5 cm vs. 5 cm; *p* = 0.000001). It was also associated with a lower 90-day SSI rate (*p* = 0.019). Conversely, the concurrent mesh subgroup had significantly more lateral hernias (*p* = 0.00001), more patients with previous abdominal surgery (*p* = 0.036), greater use of the larger needle size MSP301-5 (*p* = 0.00007), longer operative duration (*p* = 0.000007), longer length of hospital stay (*p* = 0.009) and greater length of follow-up (*p* = 0.024). All other tested variables were similar between the subgroups.

## Discussion

### Key findings

As highlighted in the review by Perez et al., most published series of Duramesh originate in the US and focus on the repair of large ventral hernias (11). There is a paucity of data describing routine indications and outcomes from centres in the UK and Europe. In this context, our study is an early experience of Duramesh from a single institution in the UK and should be interpreted as a “snapshot” of the service, rather than a controlled trial with narrowly defined indications. The broad range of indications and uses across different contamination grades in both emergency and elective settings suggests the technique is a versatile repair method.

We note that Duramesh was most utilised for small M3 hernias, an area in which there remains considerable variation in practice (15). The EHS supported Circular study will likely demonstrate that ongoing variation in the management of small umbilical hernias persists internationally (16). This variation is likely multifactorial, reflecting differing understandings of what constitutes a “small” hernia (e.g., <1 cm vs. <2 cm) and uncertainty regarding optimal repair technique. Although guidelines advocate mesh repair even for defects as small as 1 cm (17), many surgeons continue to favour suture repair, likely due to its relative technical simplicity and speed, and the challenges associated with developing a dissection plane for small defects. Our findings suggest that Duramesh may help address this clinical dilemma. It can be implanted in a manner analogous to standard suture repair while providing the potential benefits of tissue integration similar to mesh.

The short-term SSO rate of 22% in our cohort is comparable to the range reported in the literature for Duramesh closure and for mesh repair of ventral hernias (10, 18). Long-term complications such as chronic draining sinus and suture granuloma are theoretically more common with permanent materials and possibly more so with Duramesh as the knot can be thicker than those resulting from slowly absorbable monofilament suture. We note that one patient in our study developed a suture granuloma requiring explantation. However, our sample size and follow-up duration are insufficient to comment on the true incidence. Larger series, such as those from the Northwestern group, report an incidence of approximately 0.3% (12). No short-term clinical recurrences were identified following ventral hernia repair or hernia occurrence following primary laparotomy closure. However, the follow-up duration was also too short to reasonably comment on this. Thus, the effectiveness of Duramesh for preventing incisional hernia remains to be proven. While Duramesh is more expensive than conventional suture, its cost is comparable to many commercially available synthetic and composite planar meshes. As with planar mesh repair, any increased upfront cost may be offset by reduced recurrence rates, though this requires formal evaluation. Future studies should therefore incorporate cost–benefit analyses alongside clinical outcomes.

The Northwestern group recently published an algorithm where retrorectus planar mesh repair was combined with Duramesh closure of the anterior sheath (11). Whilst slightly unconventional, we too combined planar mesh repair with Duramesh closure in select cases where additional reinforcement was deemed beneficial following MDT discussion. Unsurprisingly, this subgroup had larger defects. But an interesting observation is that many were off-midline in location. In the authors experience, hernias lateral to the ambivium have minimal aponeurotic covering and Duramesh may be better suited for closure of these “muscular defects” compared to monofilament suture due to less tearing of muscle fibres. This may also explain why larger needle sizes (MSP301-5) were used in this subgroup as bites must traverse through muscle rather than a thin fascial layer.

In contrast, when Duramesh was used as a sole implant, the hernias tended to be smaller and midline in location (M1–M5). This was usually to avoid cumbersome dissection, which is supported by the significantly shorter operative duration. These cases were also associated with a lower SSI rate, but this is perhaps expected since the hernias were smaller and the total mesh burden lower. Moreover, the lower length of hospital stay and shorter follow-up duration reflect the relative simplicity of these cases, many of them day case operations with shorter convalescence period. Overall, our data suggests Duramesh repair for small midline ventral hernias could be a prime indication, but readers should be aware that these analyses were “exploratory” by nature and any differences between subgroups reflects selection bias rather than a proven difference between mesh products.

### Simple algorithm

**Figure 2:**
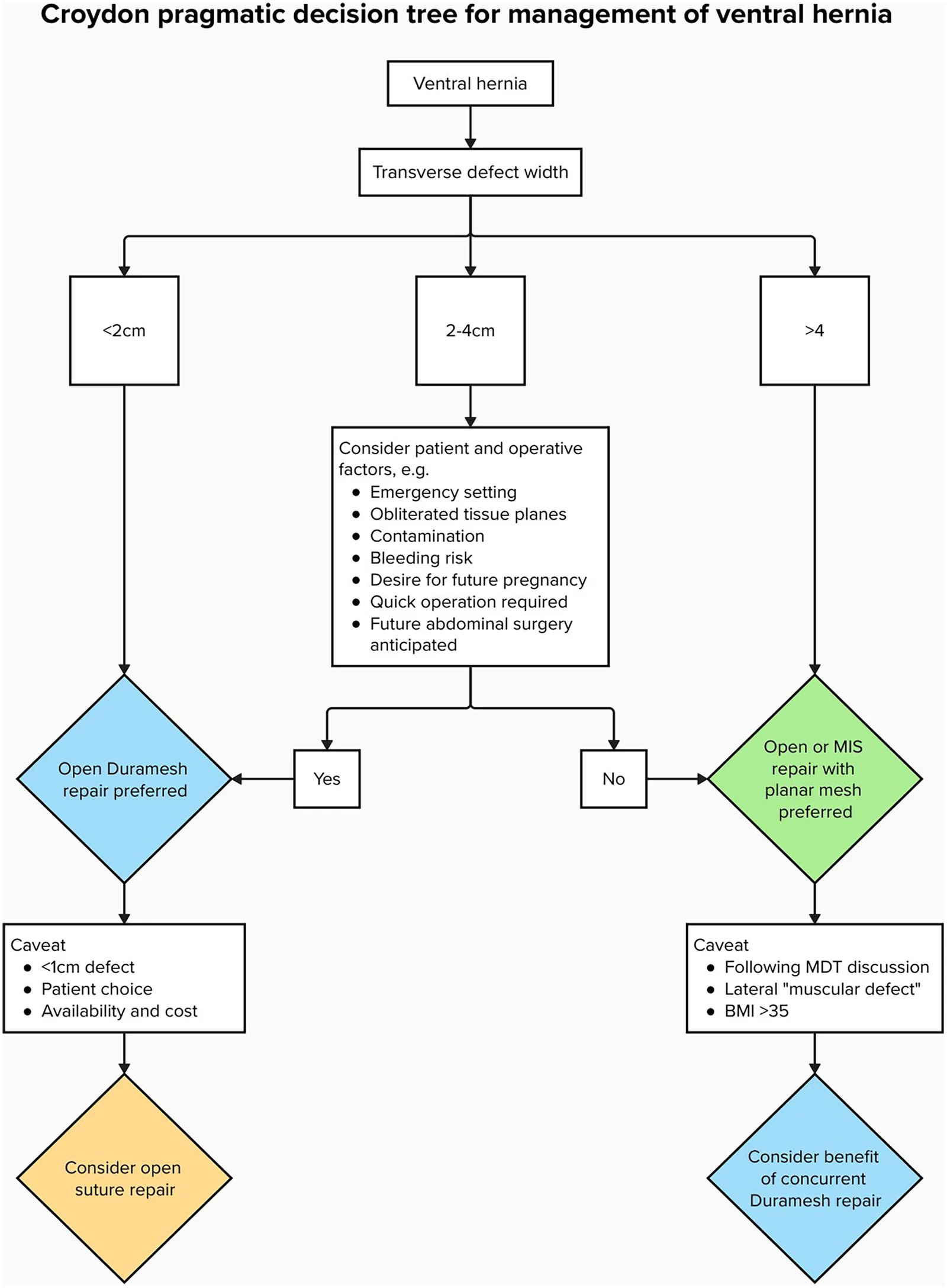
Simple clinical decision tree in use at Croydon for repair of ventral hernia. MIS, minimally invasive surgery; MDT, multidisciplinary team.

Based on our early experience, we have since developed a pragmatic decision tree for management of small to medium ventral hernias with Duramesh (Figure 2). Of note, this is not a validated algorithm but reflects current institutional practice. For defects <2 cm, open Duramesh repair has become our preferred technique. For tiny defects <1 cm at surgeon discretion or patient refusal of synthetic mesh material, suture repair can be performed instead. For defects in simple, clean cases measuring 2–4 cm, an open or minimally invasive approach with planar mesh (typically in the preperitoneal plane) remains first-line. However, in the presence of unfavourable patient or technical factors such as obliterated tissue planes, high bleeding risk, contaminated fields (CDC grade 2–3), high risk for future abdominal surgery, high risk of future pregnancy, co-morbidities necessitating a quick operation, or emergency presentation, Duramesh is used. Anecdotally, Duramesh provides a reasonably robust index repair without disrupting tissue planes in case repair of incisional hernia becomes necessary. Defects >4 cm are managed with planar mesh in line with EHS guidelines (17).

Ultimately, the decision to utilise Duramesh should be on an individual basis; however, our experience suggests it may be particularly advantageous for small hernia defects and in unfavourable conditions where planar mesh may be precluded. In select cases, we have also used Duramesh for ostomy site closure, for patients with BMI >40 as a bridging procedure to allow time for weight loss prior to definitive repair, for larger defects (4–10 cm) where abdominal compliance is good following botulinum toxin A injection, and for abdominoplasty with diastasis plication as supported by the study from Marangi et al. (19). In addition, our unit are currently using Duramesh as part of the TROCAR trial, an RCT investigating Duramesh closure of umbilical port sites (20).

### Limitations

This study has several inherent limitations. As a single-centre experience, the findings may not be generalisable to wider international practice. Furthermore, the retrospective design, short follow-up duration and relatively small sample size introduce the potential for selection bias and unmeasured confounding, and therefore the results should be interpreted with appropriate caution. We intend to collect long-term data for this series to better analyse hernia occurrence/recurrence. However, we acknowledge that this outcome was measured as symptomatic recurrence which will inevitably miss subclinical hernias. Moreover, as this was a service evaluation, imaging was not performed routinely. As a result, imaging was often not available, and if available, the timeframe from index operation was too short to determine the true hernia incidence. Thus, our result may not be representative of the long-term recurrence rate.

Whilst this series provides insight into practice within a UK-based abdominal wall unit, this remains insufficient to define optimal clinical indications for mesh suture use. In addition, although surgeons were familiar with the manufacturer's instructions, operative technique was not standardised due to the retrospective nature of the study. Variations such as interrupted vs. continuous closure, exact suture spacing and exact number of throws were not consistently recorded and therefore could not be analysed. We were also unable to assess patient-reported outcomes or perform a formal cost analysis as these data were not routinely collected. Lastly, the subgroup analyses should be interpreted as exploratory and any statistically significant result likely due to selection bias from surgical decisions guided by the decision tree. High-quality randomised controlled trials are therefore required to confirm the patient cohorts most likely to benefit from Duramesh implantation.

## Conclusion

This study demonstrates that Duramesh may be a safe and versatile adjunct for abdominal wall surgeons with acceptable early outcomes. In particular, surgeons may wish to consider Duramesh repair of small midline ventral hernias as part of their operative repertoire.

## Data Availability

The raw data supporting the conclusions of this article will be made available by the authors, without undue reservation.
